# Steering Against Wind: A New Network of NamiRNAs and Enhancers

**DOI:** 10.1016/j.gpb.2017.05.001

**Published:** 2017-09-04

**Authors:** Ying Liang, Qingping Zou, Wenqiang Yu

**Affiliations:** 1 Laboratory of RNA Epigenetics, Institutes of Biomedical Sciences & Department of General Surgery, Huashan Hospital, Cancer Metastasis Institute, Fudan University, Shanghai 200032, China; 2 Department of Biochemistry and Molecular Biology, Shanghai Medical College, MOE Key Laboratory of Metabolism and Molecular Medicine, Department of Molecular Biology, Fudan University, Shanghai 200032, China; 3 Collaborative Innovation Center for Genetics and Development, School of Life Sciences, Fudan University, Shanghai 200433, China

**Keywords:** Nuclear activating miRNAs, Tissue-specific enhancers, Transcriptional gene activation, Cell identity, Cell fate

## Abstract

MicroRNAs (miRNAs) are a class of endogenous non-coding RNAs with regulatory functions. Traditionally, miRNAs are thought to play a negative regulatory role in the cytoplasm by binding to the 3′UTR of target genes to degrade mRNA or inhibit translation. However, it remains a challenge to interpret the potential function of many miRNAs located in the nucleus. Recently, we reported a new type of miRNAs present in the nucleus, which can activate gene expression by binding to the enhancer, and named them **nuclear activating miRNAs** (NamiRNAs). The discovery of NamiRNAs showcases a complementary regulatory mechanism of miRNA, demonstrating their differential roles in the nucleus and cytoplasm. Here, we reviewed miRNAs in nucleus to better understand the function of NamiRNAs in their interactions with the enhancers. Accordingly, we propose a NamiRNA–enhancer–target gene activation network model to better understand the crosstalk between NamiRNAs and enhancers in regulating gene transcription. Moreover, we hypothesize that NamiRNAs may be involved in **cell identity** or **cell fate** determination during development, although further study is needed to elucidate the underlying mechanisms in detail.

## miRNAs and their functions

MicroRNAs (miRNAs) are small non-coding RNAs with a length of about 22 nucleotides (nt). As early as 1993, Victor Ambros identified the first miRNA in *Caenorhabditis elegans* and named it *lin-4*. He and his colleagues demonstrated that *lin-4* inhibits the expression of *lin-14* by targeting its 3′UTR region [1], [2]. Since then, this view has become widely accepted as the way in which miRNAs regulate gene expression for the last 20 years. miRNAs are found to be involved in numerous biological processes and diverse disease pathogenesis, such as cell differentiation and development, cell cycle and state, immune response, tumorigenesis, neurological disorders, and metabolic diseases [3], [4], [5], [6], [7], [8], [9], [10]. Therefore, profound understanding of miRNA regulation in all these biological activities and diseases would undoubtedly facilitate unraveling the underlying mechanisms and developing new therapeutic methods for diseases.

### Inhibitory function of miRNAs and their exceptions

Thousands of miRNAs and their target genes have been identified in mammals, and accumulating evidence shows that miRNAs are functionally distributed in numerous kinds of organisms, such as animals, plants, and microbes [7], [11], [12], [13], [14]. Mature miRNAs interact with Argonaute (AGO) and other associated proteins to form RNA-induced silencing complex (RISC), and then bind to the 3′UTR of their target genes by complementary base-pairing, resulting in the silencing of their target genes by blocking the translation process or inducing mRNA degradation [15]. Interestingly, contrary to this canonical view, several recent studies have reported that some miRNAs, such as miR-373 and miR-1, could positively regulate gene transcription through complementary promoter sequences, or directly enhance mitochondrial translation during muscle cell differentiation [16], [17], [18], [19], [20], [21]. However, it is difficult to systematically interpret the positive regulatory functions of miRNAs based on the scattered evidence.

### Evidence of miRNAs in the nucleus

Some researchers have found the presence of some miRNAs in the nucleus other than in the cytoplasm. For instance, miR-29b, which possesses a distinctive motif of a nuclear localization element, is enriched in the nucleus of Hela and NIH 3T3 cells [21]. Furthermore, next-generation sequencing studies provide clear evidence that miRNAs could be enriched in the nucleus as well [22]. In addition, several other reports consistently demonstrate that miRNAs could regulate transcription by binding to the promoter region of their target genes [16], [17], [18], [19]. For example, miR-744 induces the expression of the gene encoding mouse cyclin B1 (*Ccnb1*) by targeting its promoter region in combination with AGO1 [16]. In addition, miR-373 is also reported to induce gene expression by targeting the promoter of the gene encoding E-cadherin [17]. All of these findings indicate that miRNAs can exist in the nucleus and regulate gene expression in a manner different from how cytoplasmic miRNAs work. However, the scattered evidence in these studies is not enough to elucidate the detailed functions of nuclear miRNAs. The functions of nuclear miRNAs await further elucidation.

### Tissue-specific miRNA expression

miRNAs are distributed in a tissue-specific manner. For instance, miR-1 is specifically expressed in muscle [3]. Knockout of miR-1 in muscle could lead to the death of *Drosophila* due to disorders in muscle cell differentiation [23], whereas 50% of the miR-1-2 knockout mice died of heart developmental disorders [14]. Another tissue-specific miRNA, miR-122, is specifically expressed in liver, which may be closely related to the replication of hepatitis C virus (HCV) [24]. Although miRNAs show tissue-specific expression, it is still not clear how these miRNAs maintain their tissue-specific expression pattern. Moreover, whether these miRNAs exert their function to maintain the specificity of different tissues is also an open question.

## Enhancers and cell identity

The precise gene expression regulation during development is closely related to all kinds of complex life processes, in which most regulatory information is encoded by the enhancer [25], [26]. Enhancers, canonically termed as short noncoding DNA sequences with about 100–1000 bp in length, are a class of *cis-*regulatory elements playing pivotal roles in driving transcription independent of their locations, thus able to regulate gene expression both accurately and robustly during development [25], [26], [27]. Enhancers holds their own unique characteristics including histone modifications, p300/CREB-binding protein (CBP) co-activator binding, DNase I hypersensitivity and production of enhancer RNAs (eRNAs). Interestingly, enhancers are also involved in regulating gene expression in a tissue-specific manner, that is, the enrichment of enhancer markers shows distinct distributions in specific cell types and plays different roles in various biological activities [28].

### Markers of enhancer

Genome-wide ChIP-seq data have shown that well-defined histone modifications are enriched in enhancer regions [29]. Histone methylation, such as monomethylation of histone H3 on lysine 4 (H3K4me1), has been closely correlated with enhancer activity [29]. Another typical histone modification is H3 acetylation on lysine 27 (H3K27ac), the enrichment of which indicates a higher activity of the enhancer [30]. In addition, p300/CBP binding sites are located at the center of enhancer regions. p300 and cAMP response element-binding protein (CBP) are two crucial transcriptional co-activators that interact with a large number of transcriptional activators and catalyze the H3K27 acetylation to maintain enhancer activity [31]. Another important feature of enhancers is the enrichment of DNaseI hypersensitive sites (DHSs). DHSs are extensively distributed in almost all active *cis*-regulatory DNA regions, such as promoters, enhancers, and locus control regions [32]. The distribution pattern of DHSs can precisely describe the *cis*-regulatory compartment in chromatin. Therefore, DNase I hypersensitivity almost completely overlaps with the core region occupied by regulatory factors [32].

### eRNAs indicating enhancer activity

Enhancer also has the capability of recruiting RNAPII to transcribe enhancer RNAs (eRNAs). Most eRNAs do not undergo splicing and lack polyadenylated tails, making them very unstable [33]. Emerging evidence suggests that eRNAs are highly associated with active enhancers with increased expression of neighboring genes [34]. Global profiling also shows that eRNAs are transcribed from the center region of the enhancers, where CBP and RNAPII binding sites are located. As a result, we can predict the center region of an enhancer through eRNA production [35].

### Functional link between the enhancer markers

The four characteristics mentioned above, *i.e.*, enrichment of DHSs, H3K4me1, H3K27ac, and eRNA production, generally show the process of enhancer activation. Firstly, enhancer regions exhibit dramatic chromatin accessibility and DNaseI hypersensitivity. These chromatins with enrichment of DHSs are beneficial to RNAPII binding and eRNA production. Then, p300/CBP could interact with transcriptional activators or the general transcription machinery, including RNAPII. Finally, p300/CBP, as an acetyltransferase, can catalyze the acetylation of H3K27 when it binds to the enhancer region. Along with the occurrence of other histone modifications, a series of cognate genes are activated by enhancers.

Both enhancers and miRNAs display a pattern of tissue specificity; however, little is known about the relationship between these two “modulating elements”. Previous studies have shown that during development cell-type-specific enhancers are essential to decide cell identity and considerable disease-related genome sequences are on enhancer regions [36], [37]. Moreover, miRNAs are reported to play fundamental roles in the maintenance of cell and tissue specificity, and the occurrence of diverse diseases [9], [10], [38]. A recent study has brought strong evidence to demonstrate the interactions between miRNAs and super-enhancers. Super-enhancers can be specific marks of cell type-specific miRNAs and deletion of the constituent of super-enhancer could lead to down-regulation of miRNA expression. Moreover, this study also indicates the correlation between the function of super-enhancers with chromatin Drosha recruitment and H3K4me3 domains [39]. Accordingly, we hypothesize that these two elements may crosstalk with each other and together they play regulatory roles in the expression of genes that control cell identity and cell fate during development. A series of work was performed in our lab to evaluate the functions of both enhancers and miRNAs as active elements for mediating gene transcription. To our surprise, miRNAs are in fact able to activate gene transcription by interacting with their target enhancers [40]. This finding provides good evidence that miRNAs could serve as positive regulators in gene transcription, and potentially provide additional explanations for difficult and complex gene-related pathology.

## NamiRNA–enhancer–target gene activation network

### The origin of NamiRNAs

Since the first miRNA was discovered in 1993, more than 1800 miRNA precursors have been identified and annotated in the human genome according to the miRBase. To systematically explore the relationship between miRNA and enhancer, we analyzed the location of human miRNAs in the genome and the enrichment of the enhancer markers, such as H3K27ac and H3K4me1in 18 cell types, including GM12878, H1hESC, HSMM, HUVEC, K562, NHEK, and NHLF, which were commonly used in the ENCODE program. As a result, we obtained 1594 annotated human miRNA precursor loci, among which 1076 miRNA precursor loci partially overlapped with enhancer regions [40]. These regions are enriched within the enhancer markers: H3K27ac and H3K4me1. Furthermore, 303 highly-conserved miRNA precursors were found located in the central enhancer region with H3K27ac modification. Later, we found that some of these miRNAs, such as miR-24-1 and miR-26, are located in the nucleus and have activating functions on their neighboring genes. We named these miRNAs, which are highly associated with enhancers and located in the nucleus, as nuclear activating miRNAs (NamiRNAs).

### NamiRNAs activate neighboring gene transcription mediated by cognate enhancers

Activation of the transcription of the neighboring genes is an important function of enhancer. Based on our study, we found that NamiRNAs could have activating functions on their neighboring genes by targeting their cognate enhancers. Our previous study on miR-24-1, which is located in the enhancer region, showed that expression of its neighboring genes *FBP1* and *FANCC* was increased significantly after miR-24-1 precursor transfection, whereas transfection of miR-24-1 antagomir inhibited the expression of these genes. Similarly, transcription of genes surrounding miR-26-1, including genes encoding integrin subunit alpha 9 (*ITGA9*), carboxy-terminal domain RNA polymerase II polypeptide A small phosphatase 2-like (*CTDSPL*), villin like (*VILL*), and phospholipase C delta 1 (*PLCD1*), was also significantly activated by miR-26-1 [40]. Additionally, miR-339 and miR-3179 were also identified to activate their neighboring genes in a similar way to that of miR-24-1 and miR-26-1 in our study.

Furthermore, miRNAs could activate the cognate genes through regulating enhancer activity. In our previous work, the histone modification markers H3K27ac and H3K4me1 have been shown to be significantly enriched in miR-24-1 locus with the concurrent reduction of the inhibitory marker, H3K9me3 modification [40]. Also, the transcription factor p300, which promotes the acetylation of H3K27, is enriched at the miR-24-1 locus, indicating that miR-24-1 could promote enhancer activation in the study. In addition, eRNAs and RNAPII were significantly enriched in the enhancer region after pre-miR-24-1 transfection, suggesting a positive function of pre-miR-24-1 since eRNA transcription reflects enhancer activity [40]. In summary, these lines of evidence support that NamiRNAs are able to promote enhancers to be in an active state.

The processing of miRNA starts in the nucleus, which is closely associated with transcription by RNAPII or RNAPIII and cleavage by Drosha and Dicer to shape mature miRNA [41], [42], [43]. Both Dicer and AGO2 are indispensable in our proposed NamiRNA–enhancer–gene activation network. Moreover, enhancer disruption abolishes the NamiRNA activation function. The activation effect of pre-miR-24-1 on cognate genes was abolished in 293 T cells when the core sequence of enhancer region in miR-24-1 locus was deleted by TALEN technology [40]. This indicates that the activation of cognate genes by miR-24-1 is dependent on the intact enhancer sequence. Furthermore, NamiRNAs are also able to activate genome-wide gene transcription. Expression of 1074 genes was up-regulated when miR-24-1 was expressed ectopically based on our previous work. Interrogating a series of ChIP-seq profiles, including H3K27ac, H3K4me1, H3K9me3, and RNAPII, reveals that 3282 enhancer regions exhibit significant enrichment of active enhancer markers, such as H3K27ac, H3K4me1, and RNAPII, whereas the abundance of the negative enhancer marker H3K9me3 was reduced accordingly [40]. These results indicate that NamiRNAs could regulate enhancers even far away in the genome.

## Perspectives on the roles of NamiRNAs

### Dual functions of NamiRNAs

According to the classic view of miRNA regulation, miRNAs can block gene translation by binding to the 3′UTR of their target mRNAs. The dual presence of miR-24 transcripts in the cytoplasm and nucleus raises the question whether NamiRNAs could down-regulate gene expression by targeting their 3′UTR, aside from transcription activation. The answer is yes. Microarray analysis indicates that miR-24-1 down regulates the expression of a group of genes with miR-24-1binding sites present in their 3′UTR, suggesting that these genes may be negatively regulated by miR-24-1 [40].

**Figure 1:**
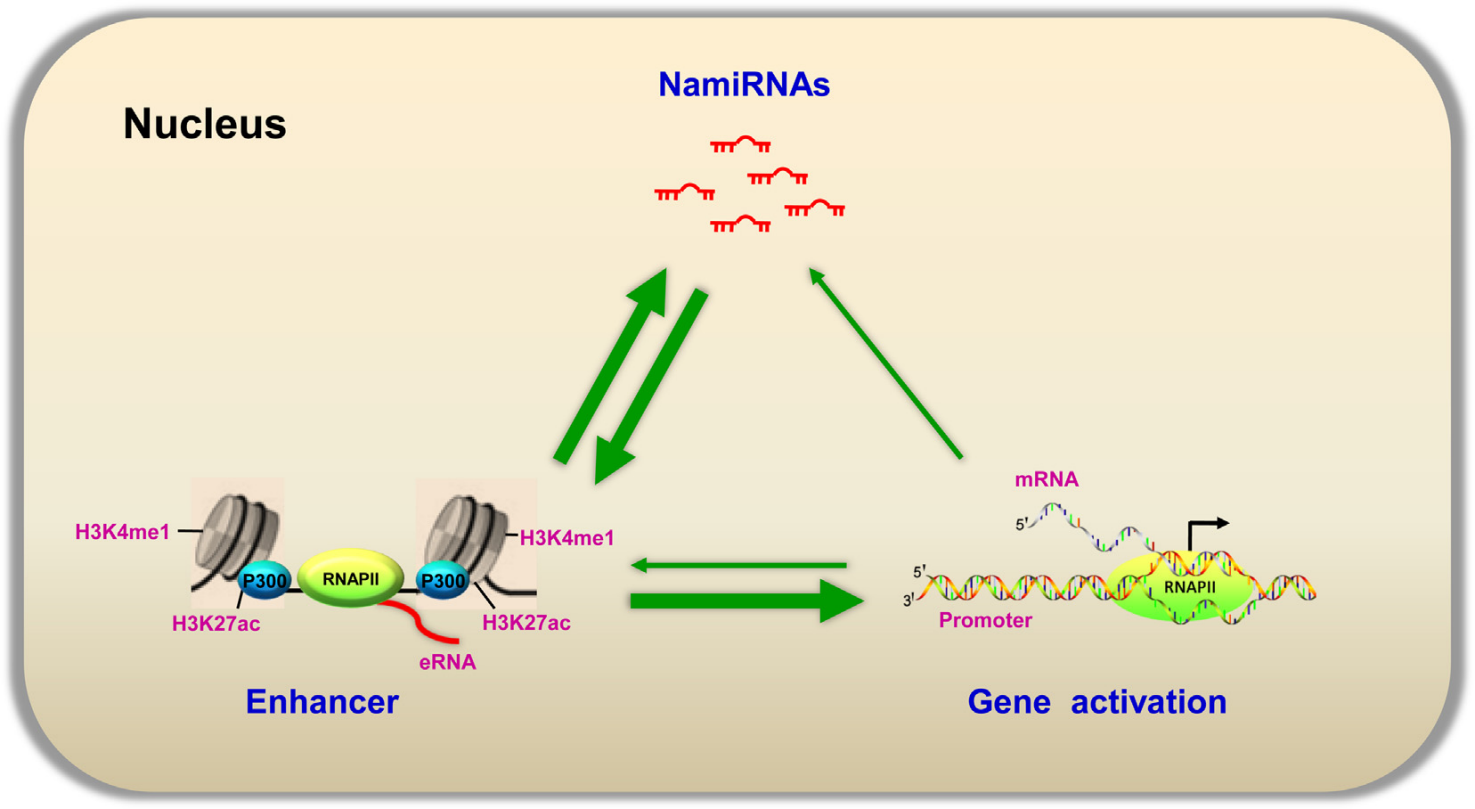
**Schematic diagram showing a network for NamiRNAs, enhancers, and gene activation** Active enhancers have some features, including H3K4me1, H3K27ac, p300/CBP co-activator binding, DNase I hypersensitivity, and production of eRNAs. NamiRNAs could interact with and activate the enhancers. Meanwhile, enhancers could also activate the expression of endogenous NamiRNAs and proximal genes. Thus, enhancers and NamiRNAs form a positive feedback network. Tissue-specific transcription factors may sometimes regulate the expression of miRNAs by targeting their promoters, or participate in the process of enhancer activation. In the figure, the thin green arrow indicates that the encoded transcription factors could regulate the expression of miRNAs or the activation of enhancer; the thick green arrow indicates that enhancer and NamiRNA could regulate the expression of target genes mutually or individually. RNAPII, RNA polymerase II.

Our previous study unexpectedly reveals that miRNAs, other than inhibiting gene expression, can also function as activating factor in the nucleus in combination with their cognate enhancers. This leads us to hypothesize that miRNAs play dual functions in gene regulation in the cytoplasm and the nucleus. That is, miRNAs have an inhibitory function in the cytoplasm and an activating function in the nucleus, respectively. We thus put forward a functional network of NamiRNAs and enhancers to demonstrate their interactions for the positive regulation of gene transcription. This NamiRNA–enhancer–target gene activation network describes the novel function of miRNA in the nucleus (Figure 1). According to our model, NamiRNAs could interact with the enhancer, promote the enrichment of active enhancer markers, such as H3K27ac and H3K4me1, and alter chromatin status in the enhancer regions, thus activating the cognate gene transcription at genome-wide scale. Enhancers could also activate the expression of endogenous NamiRNAs and proximal genes. Therefore, both enhancers and NamiRNAs form a positive feedback network.

Our model could help explain why after exogenous miRNA transfection, we often observed downregulated expression of some genes with upregulated expression of other genes in RNA-seq data. Previously, we only paid attention to down-regulated genes but ignored the up-regulated ones to reconcile with the accepted notion for miRNA regulation. Now, by taking into account our new NamiRNA-enhancer-target gene activation network model, miRNA studies could be substantially broadened to elucidate various physiological and pathological phenomena occurring during cell differentiation, development and tumor genesis and metastasis.

### NamiRNAs may be involved in cell identity or cell fate decision

Cell identity and cell fate reprogramming are regulated by integral gene regulatory networks, comprising transcription factors, epigenomic regulators, and non-coding RNAs, such as miRNAs [44], [45], [46]. In recent years, great achievements have been made in explaining how miRNAs play crucial roles in controlling cell fate during development and maintaining cell identity. For instance, miR-7 is reported to play a pivotal role in the acquisition of the pancreatic β-cell fate during development and maintenance of the mature pancreatic β-cell identity [47]. As a subclass of CD4^+^T helper (Th) cells, T follicular helper (Tfh) cells can mediate the formation and maintenance of germinal centers and assist antigen-specific B cells to defend infections [48]. miRNAs such as the miR-17∼92 clusters and miR-155 are involved in shaping Th cell identity, while miR-146a is repressed in the development and expansion of Tfh cells during differentiation, suggesting that different miRNAs may function in different manners in the progress of cell differentiation [48], [49]. Unfortunately, how miRNAs exert their functions during cell differentiation and cell fate reprogramming remains elusive. The molecular mechanism underlying the aforementioned phenomena may be illustrated more clearly if the NamiRNA–enhancer–target gene activation network could be taken into account to investigate the “selectively ignored” up-regulated genes. In the future, scientists could consider this model to explain interactions between the enhancers and miRNAs, thus broadening our understanding of cell identity mechanisms for a more complete picture.

As a group of classic *cis*-regulatory elements, enhancers can drive cell-type-specific gene expression related to cell identity and cell fate reprogramming. Their key roles in the regulation of pluripotent stem cells (PSCs) and embryonic stem cells (ESCs) have been verified in several studies. For example, super-enhancers, which are made up by a cluster of transcriptional enhancers, can regulate the expression of several key genes that controls cell identity in PSCs during the differentiation from stem cells into terminal cells [50]. Another case is that the IgH intronic enhancer Eμ, which is a combination of a 220-bp core region and two flanking matrix attachment regions. Eμ influences VDJ rearrangement and thus takes part in B cell fate decision, and a deletion of this core regulatory region leads to both a decline in the number of pre-B-cells and newly shaped B-cells [51]. In addition, a recent study has demonstrated that enhancers primed by H3K4 methyltransferase myeloid/lymphoid or mixed-lineage leukemia 4 (MLL4) play essential roles in cell fate transition during differentiation in mouse ESCs [52]. Still, how enhancers are regulated and activated mechanistically still awaits further investigation. Notably, the recent study of Sharp and his colleagues have also shed light on the association between miRNAs and enhancers. They showed that super-enhancers, as an emerging subclass of regulatory elements controlling cell identity, in company with broad H3K4me3 domains, regulate the expression and processing of master miRNAs to establish a cell-type-specific pattern [39]. Hopefully, applying NamiRNA–enhancer–target gene activation network to the cell fate decision or cell identity-related studies, together with cell differentiation elements, will help to elucidate the unexplained steps in these processes.

**Figure 2:**
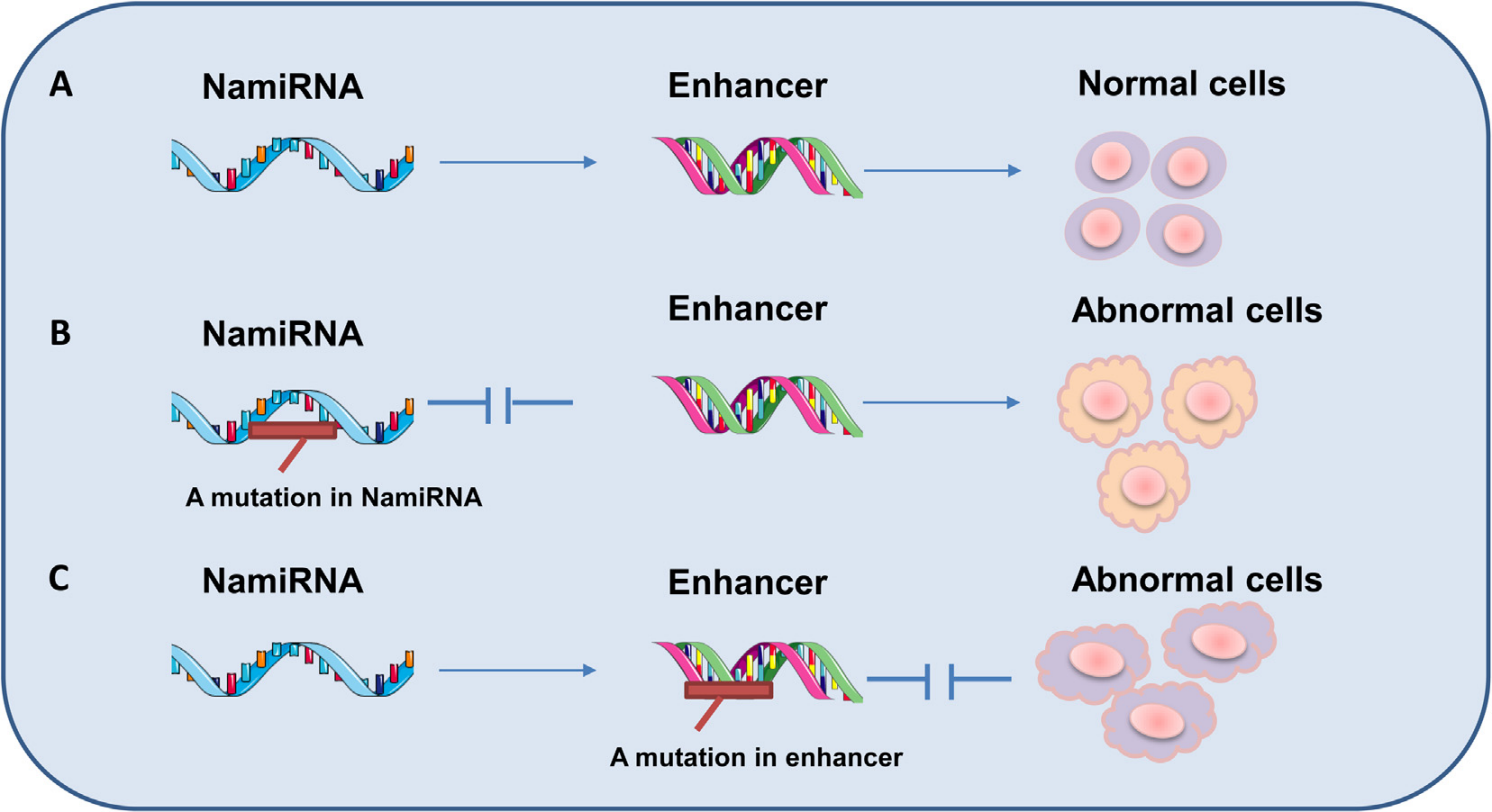
**NamiRNAs and enhancers are essential to regulate gene expression in controlling cell fate** We propose a NamiRNA–enhancer–cell fate decision network to demonstrate the interactions between the enhancers and NamiRNAs in cell identity determination. **A.** Under normal conditions, NamiRNAs interact with enhancers to activate the expression of genes related to cell identity, leading to normal cell differentiation. **B.** Mutations in NamiRNAs may result in the silence or dysfunction of the cell identity-related genes, thus leading normal cells to death or an abnormal state. **C**. Similarly, mutations in enhancer regions may also contribute to the death or change into an abnormal state for the normal cells.

So far, efforts made in our lab have been focused to better understand the connection between NamiRNAs and enhancers, as well as the function of NamiRNAs in cell differentiation and cell fate reprogramming. Building upon NamiRNA–enhancer–target gene activation network model, we propose a NamiRNA–enhancer–cell identity/cell fate decision model, that is, NamiRNAs can activate gene expression by binding to the targeted enhancers and playing regulatory roles during cell development, thereby enabling the control of cell fate or cell identity. Mutations in NamiRNAs or enhancer regions could cause dysfunction of cell fate transition and cell identity controlling, thus leading to cell death or tissue lesions (Figure 2).

However, more systematic studies are needed for a comprehensive understanding of the function of NamiRNAs related to cell identify and cell fate reprogramming. It remains to be answered whether NamiRNAs play a much broader role in cell identity controlling or cell fate transition by recruiting other regulatory factors such as transcriptional factors, miRNA processing elements like DGCR8, or any other functional enzymes besides the aforementioned p300, AGO2, Dicer, and eRNAs. Although our lab has found that NamiRNAs interact with the enhancer regions, more evidence is needed to demonstrate whether NamiRNAs bind to enhancers directly or in an indirect manner. This would help to better understand the molecular mechanism underlying the NamiRNA–enhancer–target gene activation network. It is also important to point out that researchers interested in NamiRNAs should pay attention to see how AGO2 plays their regulatory function on NamiRNAs and enhancer regions both *in vivo* and *in vitro* for future studies. We believe that findings from our and other labs about miRNAs and enhancers will broaden miRNA research and lead to new clues for further investigations by a new view of the previously-overlooked side of miRNA regulation.

## Competing interests

The authors declare that no potential conflicts of interest exist.
